# A facile preparation of FePt-loaded few-layer MoS_2_ nanosheets nanocomposites (F-MoS_2_-FePt NCs) and their application for colorimetric detection of H_2_O_2_ in living cells

**DOI:** 10.1186/s12951-019-0465-3

**Published:** 2019-03-13

**Authors:** Zunfu Hu, Zhichao Dai, Xiaowei Hu, Baochan Yang, Qingyun Liu, Chuanhui Gao, Xiuwen Zheng, Yueqin Yu

**Affiliations:** 1 Collage of Chemistry and Molecular Engineering, Qingdao University of Science and Technology, Qingdao, China; 2 Key Laboratory of Functional Nanomaterials and Technology in Universities of Shandong, Linyi University, Linyi, China; 3 School of Materials Science and Engineering, Linyi University, Linyi, 276000 China; 4 School of Chemistry and Environmental Engineering, Shandong University of Science and Technology, Qingdao, 266510 People’s Republic of China

**Keywords:** Few-layer MoS_2_ nanosheets, FePt, Colorimetric, H_2_O_2_, Intracellular H_2_O_2_

## Abstract

**Background:**

Rapid and sensitive detection of H_2_O_2_ especially endogenous H_2_O_2_ is of great importance for series of industries including disease diagnosis and therapy. In this work, uniform FePt nanoparticles are successfully anchored onto Few-layer molybdenum disulfide nanosheets (F-MoS_2_ NSs). The powder X-ray diffraction, transmission electron microscopy, UV–Vis spectra and atomic force microscopy were employed to confirm the structure of the obtained nanocomposites (F-MoS_2_-FePt NCs). The prepared nanocomposites show efficient peroxidase-like catalytic activities verified by catalyzing the peroxidation substrate 4,4′-diamino-3,3′,5,5′-tetramethylbiphenyl (TMB) with the existence of H_2_O_2_.

**Results:**

The optimal conditions of the constructed colorimetric sensing platform is proved as 35 °C and pH 4.2. Under optimal catalytic conditions, the detection limit for H_2_O_2_ detection reaches 2.24 μM and the linear ranger is 8 μM to 300 μM. Furthermore, the proposed colorimetric sensing platform was successfully utilized to detect the intracellular H_2_O_2_ of cancer cells (MCF-7).

**Conclusions:**

These findings indicated that the F-MoS_2_-FePt-TMB-H_2_O_2_ system provides a potential sensing platform for hydrogen peroxide monitoring in living cells.

**Electronic supplementary material:**

The online version of this article (10.1186/s12951-019-0465-3) contains supplementary material, which is available to authorized users.

## Background

Hydrogen peroxide (H_2_O_2_) takes an essential position in many biochemical reactions, such as metabolism of proteins and carbohydrates. Furthermore, it can be used as a significant indicator of the occurrence of many serious disease especially cancer [1, 2]. Consequently, a sensitive, cost-effective, rapid and easy operation method for H_2_O_2_ determination would be demanded for bioassays and environmental applications [3]. Up to now, several techniques for H_2_O_2_ determination, such as chromatography [4], chemiluminescence, electrochemistry [5, 6] and colorimetric method [7], have been reported. Among these techniques, colorimetric route has several outstanding advantages, including visibility, low cost, easy automation, portability and operation convenience [8]. Although enormous progresses have been made, sensitive and rapid detection of H_2_O_2_ still remains highly need. Recently, due to its high selectivity, many nanometerials were employed to construct colorimetric sensors to detect H_2_O_2_.

Conventional enzymes are especially effective when catalyze series of reactions under mild conditions. However, conventional enzymes have rigorous limitations in practical use because they usually show insufficient stability in cruel conditions, additionally, they are hard to purify and preserve [9]. Therefore, over the past few decades, an explosion of interests have been drawn to study enzyme-mimic materials aiming to get high efficiency without the mentioned shortcomings. To date, versatile nanomaterials, such as CoS NPs [10], Fe_3_O_4_ NPs [11, 12], Copper nanoclusters [7], metal–organic framework [13], WS_2_ nanosheets [14], graphene oxide [15, 16], and kinds of metals [5, 17, 18] are used to fabricate nano-enzymes and exhibit effective catalytic activities suggesting prospective potentials in numerous bio-field, accompanied by series of advantages, including cost-effective, simple process, readily available raw materials, easy purification of products, low cost and long guarantee period [19, 20].

Molybdenum disulfide (MoS_2_), with a graphene-like lamellar structure, is composed of S–Mo–S sandwich structure and held by weak van der Waals forces. The few-layer MoS_2_ nanosheets (F-MoS_2_ NSs) with excellent 2D structure possess a direct bandgap of 1.8 eV, which is much higher than the indirect bandgap in bulk MoS_2_ NSs (1.2 eV) [21]. Hence, great efforts have been devoted to prepare few-layer MoS_2_ NSs and they are applied in sensing, catalysis, supercapacitors and so on [22–27]. Furthermore, based on its super-large specific surface areas and abundant active edges, MoS_2_ NSs have been utilized as base material to integrate with series of nanomaterials to further improve their catalytic performance [28]. A variety of monometallic nanoparticles (MNPs), such as Ag [29, 30], Pd [31], Pt [27], Au [32, 33] and Co NPs [34] have been successfully decorated on 2D MoS_2_ NSs. The obtained MoS_2_-MNPs can enhance their intrinsic properties. However, it is extremely difficult to further enhance the catalytic efficiency. Therefore, bimetallic nanoparticles (BNPs) were developed to improve the catalytic abilities [35–41].

**Scheme 1:**
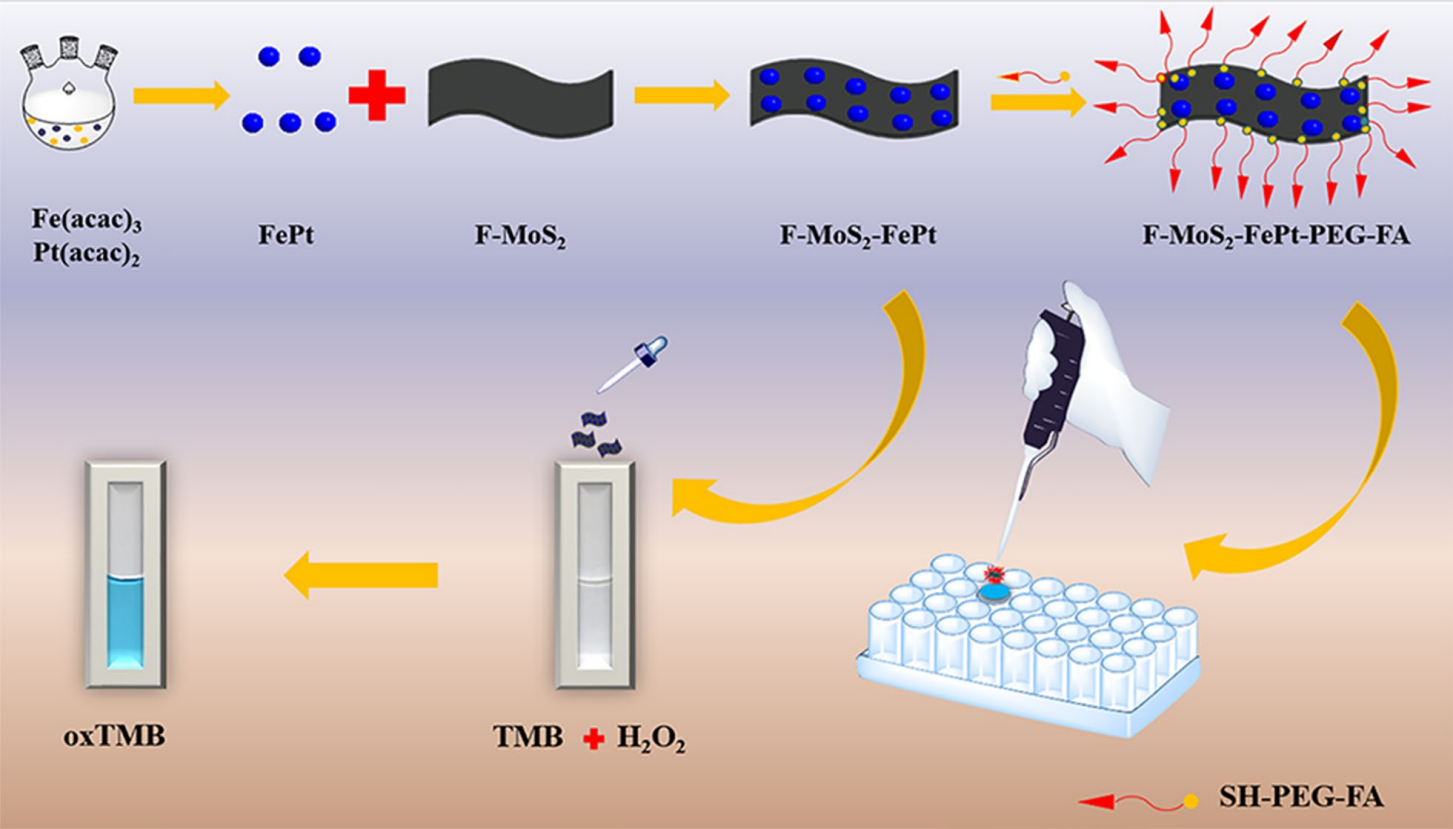
Synthesis of few-layer MoS_2_-FePt and schematic representation of the prepared colorimetric sensing platform for H_2_O_2_ detection in vivo

Platinum-based BNPs have been widely used as sensing materials in non-enzymatic H_2_O_2_ sensing platforms and they show excellent electronic and catalytic properties. Until now, few attempts have been made to study the peroxidase-like catalytic ability of FePt NPs. Therefore, both MoS_2_ and FePt NPs are expected to be employed together for the development of colorimetric sensor for H_2_O_2_ detection. In this work, few-layer MoS_2_ NSs (F-MoS_2_) loaded uniformly FePt NPs are prepared and the catalytic activity of the obtained NCs are systematically studied. Scheme 1 illustrates the technical route to prepare the NCs and the method to detect H_2_O_2_.

## Results and discussion

### Characterization of FePt nanoparticles, F-MoS_2_ NSs and F-MoS_2_-FePt NCs

The way to prepare F-MoS_2_-FePt NCs is depicted in Scheme 1, while the experiments details are described in the experimental section. In this work, few-layer MoS_2_ NSs (F-MoS_2_) are obtained by exfoliating bulk MoS_2_ via lithium intercalation–exfoliation. To investigate the thickness of the as-prepared MoS_2_ NSs, atomic force microscopy (AFM) is utilized to measure as-prepared MoS_2_ NSs. As shown in Additional file 1: Figure S1, the altitude of as-prepared MoS_2_ NSs is around 2 nm, implying as-prepared MoS_2_ NSs have 2–3 layers [25, 42].

**Fig. 1:**
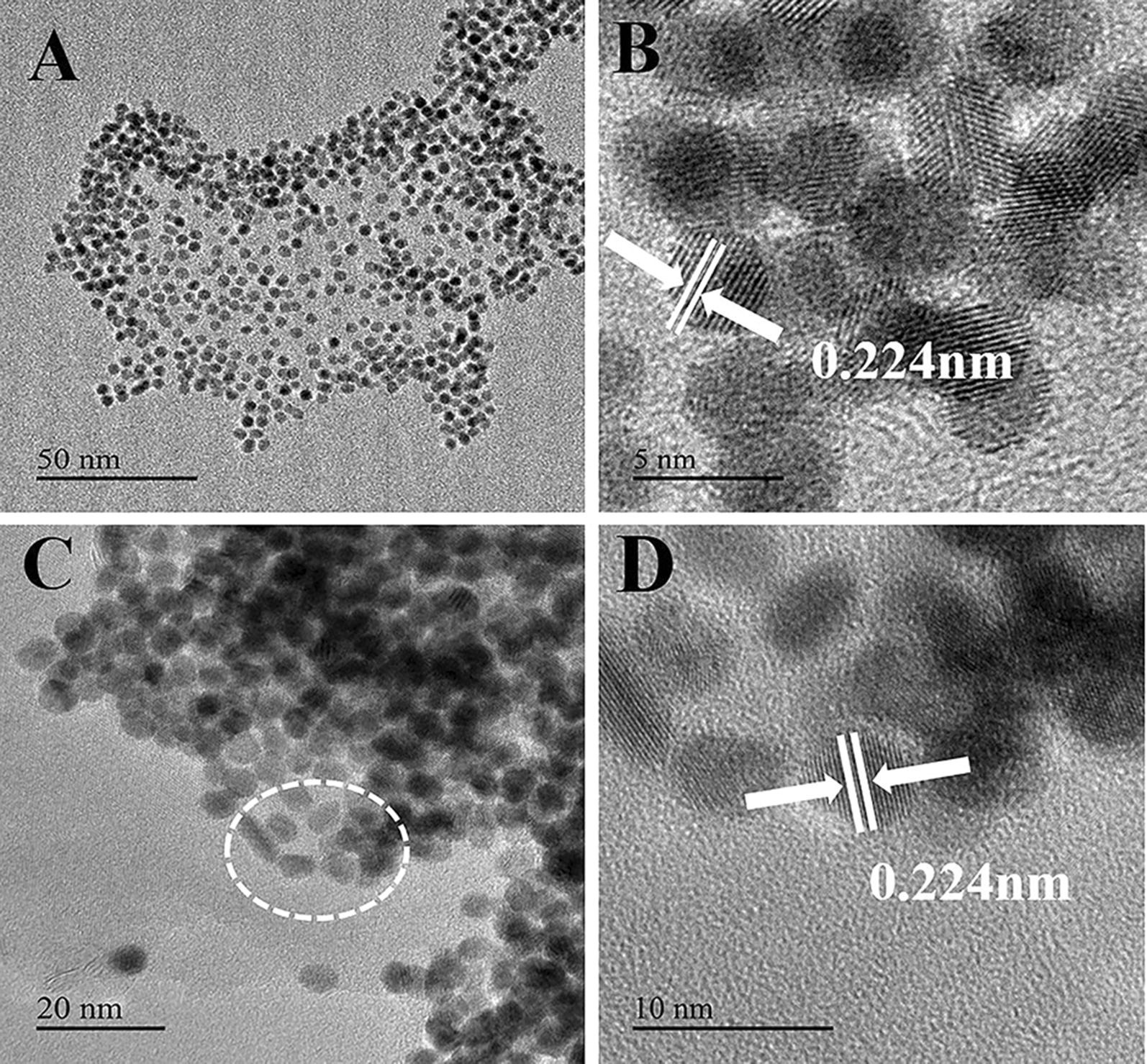
TEM image of FePt NPs (**A**), HRTEM image of FePt NPs (**B**), TEM image of F-MoS_2_-FePt (**C**) and HRTEM image of F-MoS_2_-FePt (**D**)

Then TEM is employed to characterize the obtained nanomaterials. As illustrated in Fig. 1A, FePt NPs show uniform spherical morphology and the diameter is 4 nm. The lattice fringes of as-prepared FePt NPs can be seen obviously in Fig. 1B, the adjacent fringe spacing is about 0.224 nm, corresponding to the (111) lattice planes of FePt [43, 44]. To equally disperse FePt NPs on the surface of F-MoS_2_ NSs, the obtained FePt NPs are firstly transformed from the organic phase to aqueous phase via ligand exchange. The zeta potential of FePt-DMSA is − 25 mV, indicating the successful modification of FePt NPs by dimercaptosuccinic acid (DMSA) (Additional file 1: Figure S2). After modified with DMSA, FePt NPs can be well-dispersed in water. Then, the thiolated FePt NPs could be easily anchored on the defect-rich edge sites of F-MoS_2_ NSs. As shown in Fig. 1C, FePt NPs are successfully anchored onto the surface of F-MoS_2_ NSs. Bulk MoS_2_ NSs are thicker and more visible than the exfoliated F-MoS_2_ NSs, as shown in Additional file 1: Figure S3. The lattice fringe spacing of the loaded nanoparticles is also 0.224 nm, similar to the monodispersed FePt NPs, as illustrated in Fig. 1D.

**Fig. 2:**
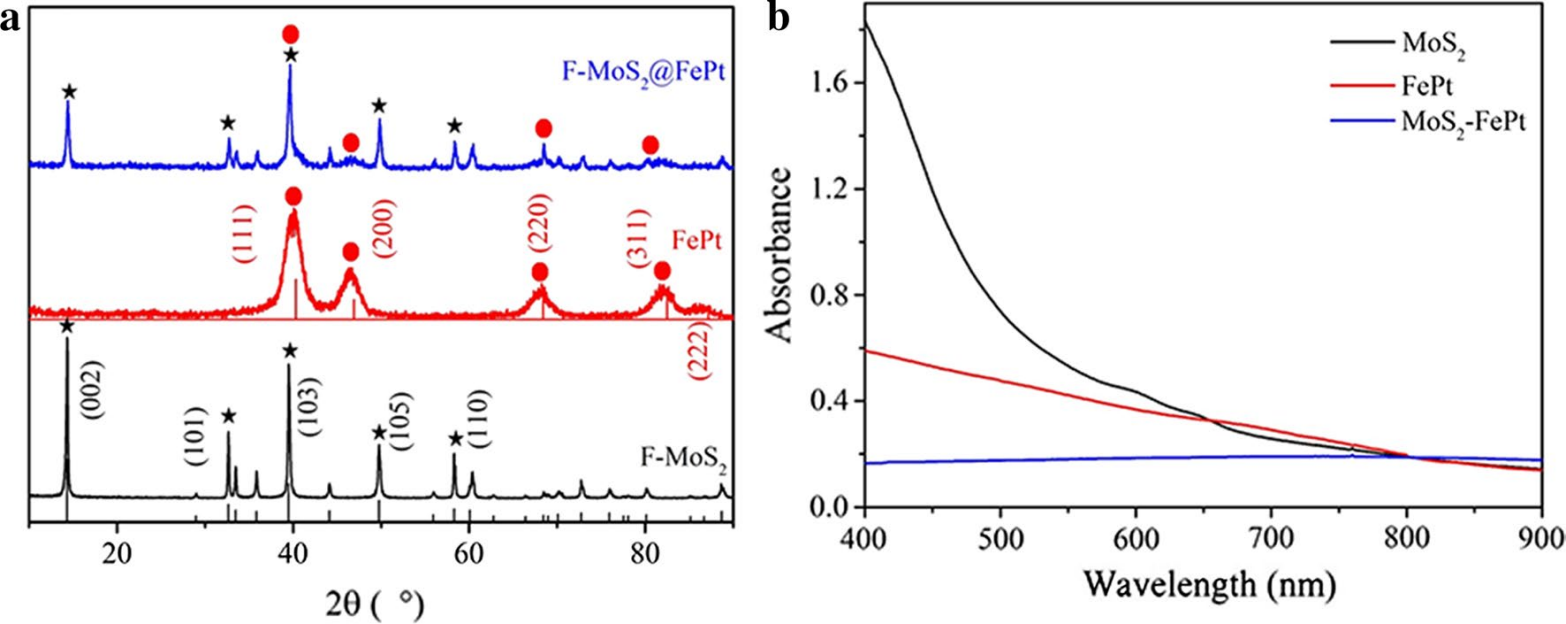
**a** XRD patterns of MoS_2_ (black line), FePt (red line) and F-MoS_2_-FePt NCs (blue line). **b** UV–Vis spectra of MoS_2_ (black line), FePt (red line) and F-MoS_2_-FePt (blue line)

Powder X-ray diffraction is utilized to further confirm the crystalline structure of as-prepared F-MoS_2_ NSs, FePt NPs and F-MoS_2_-FePt NCs. The data of X-ray diffraction are displayed in Fig. 2a. The exfoliated F-MoS_2_ NSs exhibit series of highlighted peaks in accordance with the reported ultrathin MoS_2_ nanosheets [33]. The XRD spectra of FePt NPs show four peaks in accordance with the reported FePt NPs [45]. Nearly all peaks of FePt NPs and F-MoS_2_ NSs are found in the XRD patterns of F-MoS_2_-FePt NCs, shown in Fig. 2a. All data show that F-MoS_2_-FePt NCs are successfully prepared. Furthermore, UV–Vis spectra are employed to characterize the obtained nanomaterials. As presented in Fig. 2b, after modified with FePt NPs, the strong absorbance of the exfoliated F-MoS_2_ NSs is covered by sufficient FePt NPs, which show no obvious absorbance band from 400 to 900 nm. These results demonstrate that FePt NPs is successfully anchored on the surface of F-MoS_2_ NSs.

### Peroxidase-like activity of the obtained FePt, F-MoS_2_ NSs and F-MoS_2_-FePt NCs

**Fig. 3:**
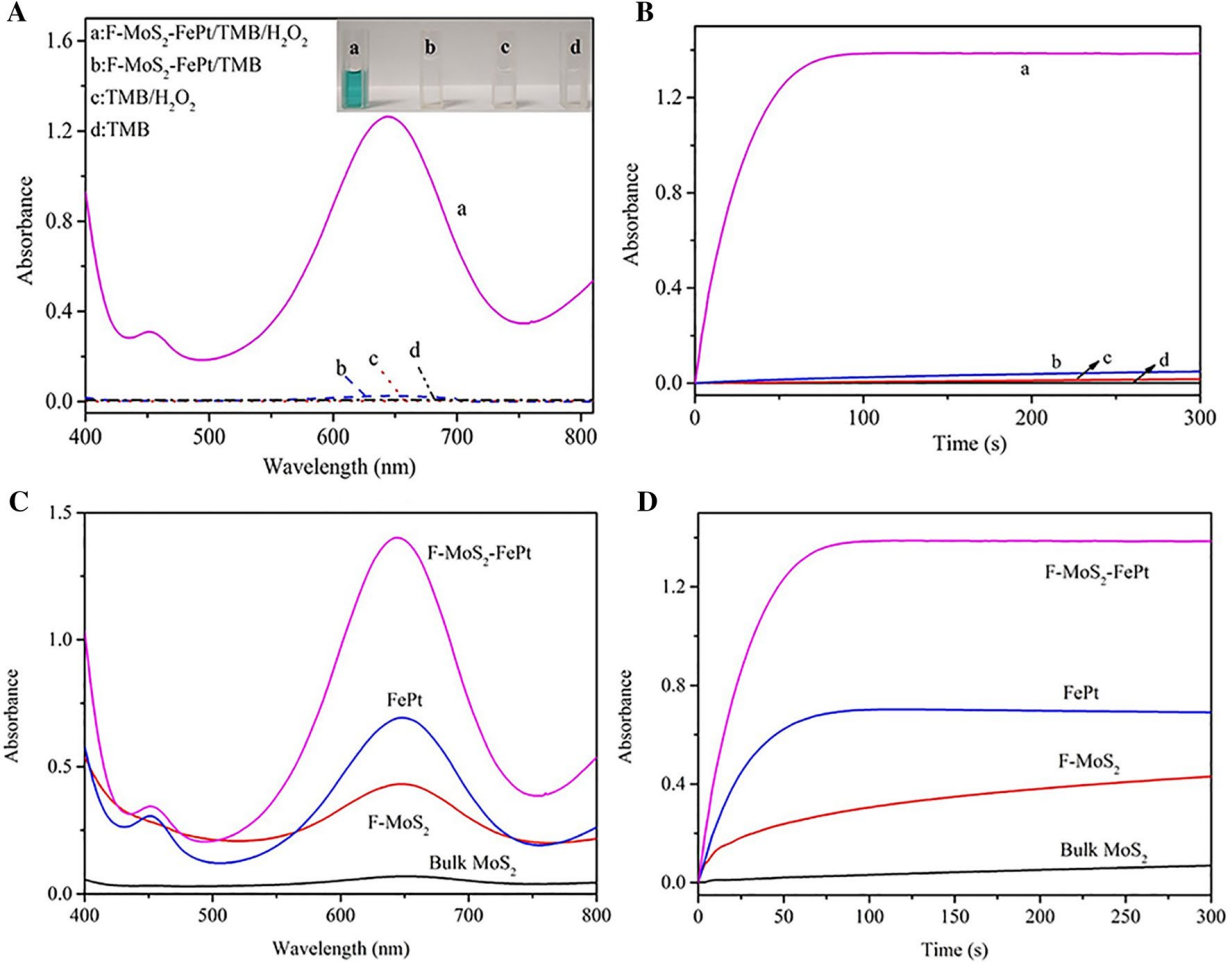
**A** The investigation of peroxidase-like activity. (a) F-MoS_2_-FePt NCs/H_2_O_2_/TMB; (b) F-MoS_2_-FePt NCs/TMB; (c) H_2_O_2_/TMB; (d) TMB. **B** Time-dependent ultraviolet absorbance changes at 652 nm of these experiments. **C** The UV–Vis spectra of these obtained nano-enzymes. **D** Time-dependent ultraviolet absorbance changes at 652 nm of these obtained nano-enzymes

To investigate the peroxidase-like catalytic activities of F-MoS_2_-FePt NCs, 4,4′-diamino-3,3′,5,5′-tetramethylbiphenyl (TMB) is selected as chromogenic substrate to induce the color reaction. As depicted in Fig. 3A, a prominent absorption peak of the oxidation products at 652 nm is observed, while the other three systems do not have any well-developed peaks ranging from 400 to 800 nm. As shown in the inset of Fig. 3A, in the presence of F-MoS_2_-FePt NCs and H_2_O_2_, the TMB solution turns blue promptly. However, the TMB solution remains colorless in the absence of either H_2_O_2_ or F-MoS_2_-FePt NCs. As illustrated in Fig. 3B, the absorbance of F-MoS_2_-FePt/TMB/H_2_O_2_ at 652 nm climbs rapidly and maintains constantly within 100 s, indicating TMB could be oxidized rapidly, while the absorbance of the reference experiment remains unchanged. The results prove that the obtained F-MoS_2_-FePt NCs possess efficient peroxidase-like catalytic activity.

UV spectrum is utilized to estimate the catalytic activities of F-MoS_2_-FePt NCs, bulk MoS_2_ NSs, F-MoS_2_ NSs and FePt NPs. As illustrated in Fig. 3C, the absorbance of F-MoS_2_-FePt NCs reaches the highest value among all the materials. Moreover, the absorbance of F-MoS_2_ NSs is much higher than bulk MoS_2_ NSs, which is attributed to the higher specific surface area and more exposed active sites. Furthermore, the time-dependent mode of the UV–Vis spectra at 652 nm for these materials is also investigated. As depicted in Fig. 3D, the UV spectra of F-MoS_2_-FePt NCs at 652 nm reach the balance within 100 s and the highest value is obtained, which indicates the strong synergistic effect between F-MoS_2_ NSs and FePt NPs [46].

**Fig. 4:**
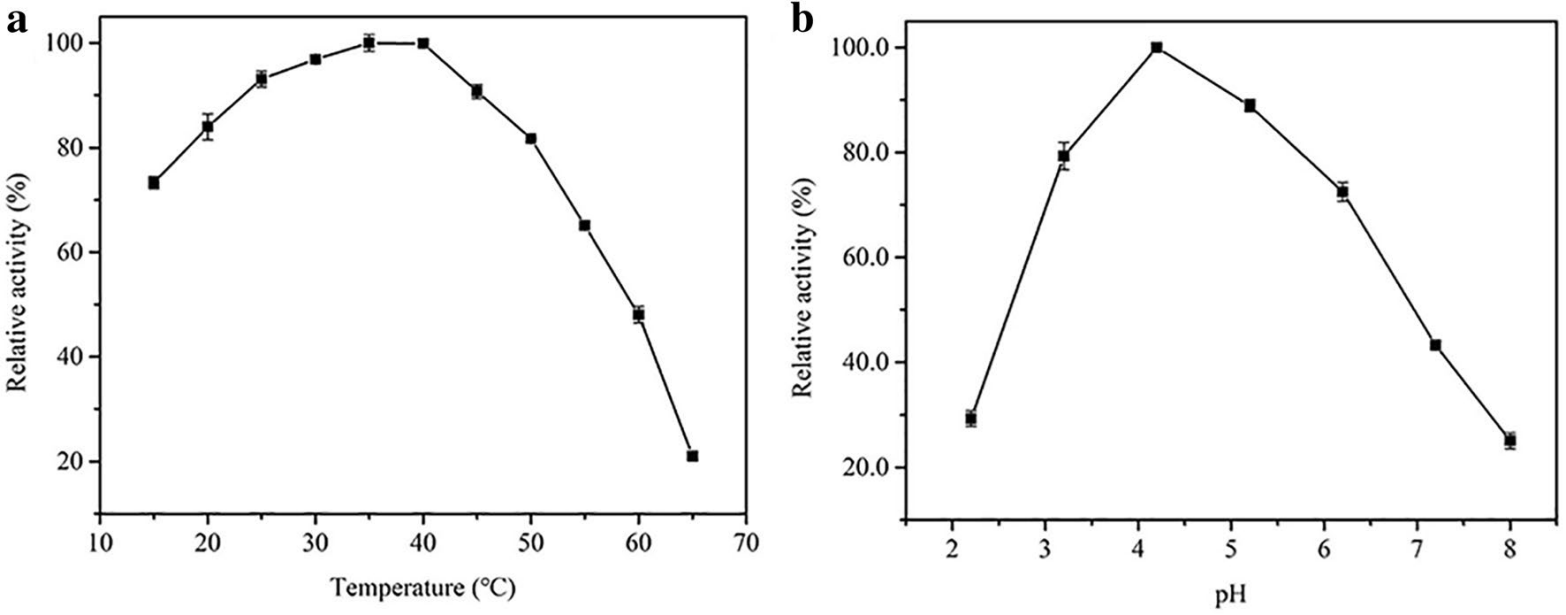
The effects of temperature (**a**) and pH (**b**) on the catalytic activity of F-MoS_2_-FePt NCs. The reaction conditions are shown as follows: 1.4 mL CPBS (pH 4.2), 200 μL TMB (1 mM), 200 μL F-MoS_2_-FePt HNPs (20 μL mL^−1^) and 200 μL H_2_O_2_ (0.25 M), the pH of the solution varies from 2.2 to 8

Similar to other enzyme-mimic systems, temperature and pH play vital roles in the catalytic activities of F-MoS_2_-FePt NCs. As depicted in Fig. 4a, the absorption at 652 nm keeps relatively high between 20 and 50 °C and reaches the maximum value at about 40 °C. Similarly, the influence of the pH of the TMB solution is also investigated as the pH varies from 2.2 to 8. As depicted in Fig. 4b, higher UV–Vis absorption is acquired when the TMB solution is kept weakly acidic, which indicates that the weakly acidic environment would be beneficial to the oxidation of TMB. However, when the solution is kept neutral or basic, the UV–Vis absorption is relatively low, which is mainly attributed to the reason that under basic solution, more OH^−^ groups are absorbed on F-MoS_2_-FePt NCs, occupying active sites of F-MoS_2_-FePt NCs for the further reaction with H_2_O_2_ [47]. In summary, 35 °C and weakly acidic condition (pH = 4.2) are chosen as the optimum conditions.

### Kinetic investigation of F-MoS_2_-FePt NCs as peroxidase mimics

**Fig. 5:**
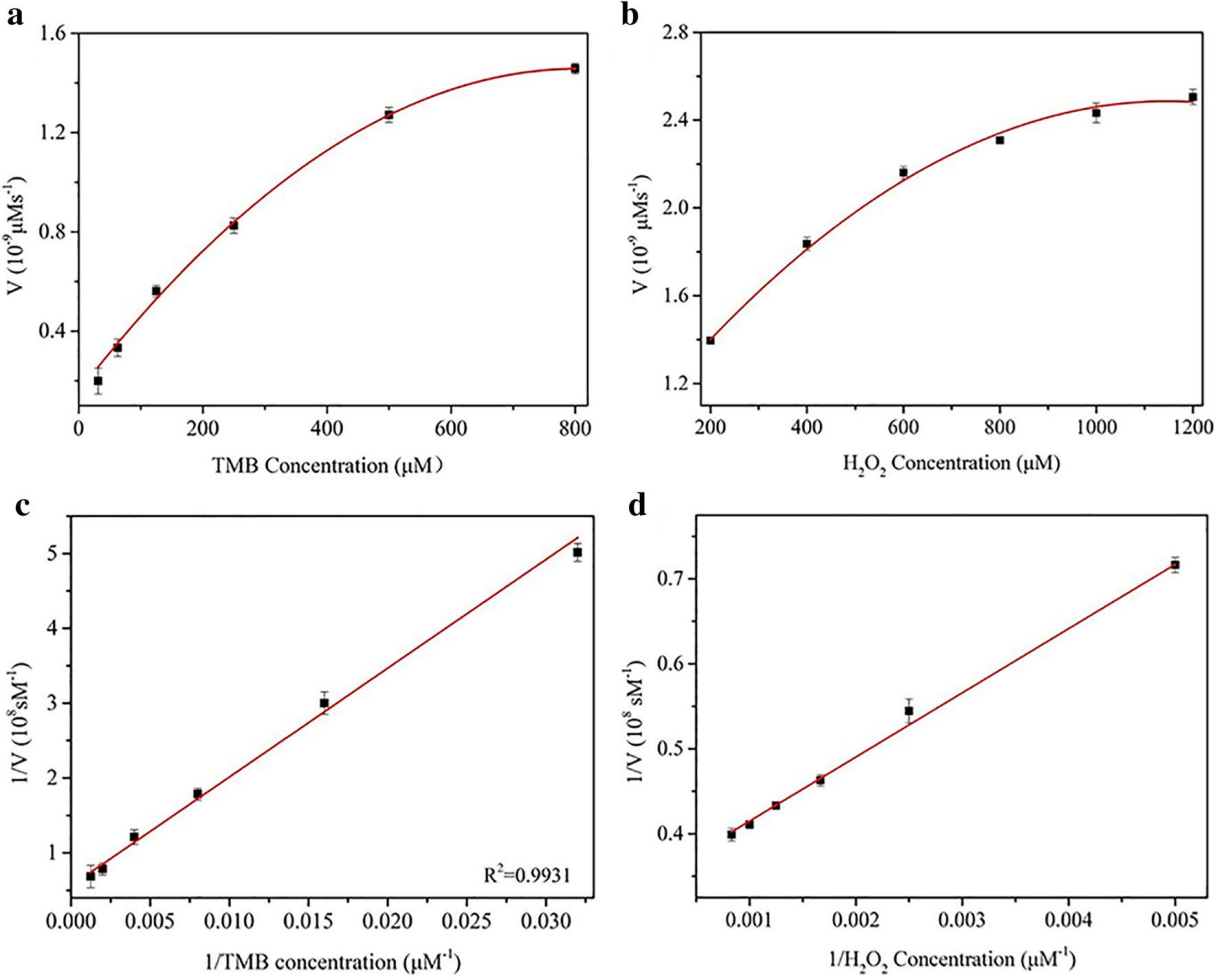
Kinetic curves of F-MoS_2_-FePt NCs. The concentration of TMB (**a**) and H_2_O_2_ (**b**) are varied. Double reciprocal plots F-MoS_2_-FePt NCs by changing the concentration of TMB (**c**) and H_2_O_2_ (**d**)

Under optimal conditions, TMB and H_2_O_2_ are chosen as the substrates to study the steady-state kinetic of the prepared F-MoS_2_-FePt NCs. As illustrated in Fig. 5a, b, when TMB or H_2_O_2_ is catalyzed in certain concentration range, normative Michaels-Menten curves are acquired. Michaels-Menten constant (*K*_m_), which represents the affinity between substrates and catalyst, and the initial reaction velocity (*V*_max_) are reckoned from the L-B plot, the results are displayed in Fig. 5c, d. Based on the calculation, for the obtained F-MoS_2_-FePt NCs, the *K*_m_ value is 0.2225 mM and the relevant *V*_max_ is 2.9458 × 10^−8^ M s^−1^. Correspondingly, the *K*_m_ and *V*_max_ values with TMB are 0.4283 mM and 1.7857 × 10^−8^M s^−1^. Compared with other reported artificial enzymes and Horseradish peroxidase, the *K*_m_ and *V*_max_ are much smaller, which represents higher affinity between F-MoS_2_-FePt NCs and the substrates (H_2_O_2_ and TMB) [40, 48–50].

### Catalytic mechanism

**Fig. 6:**
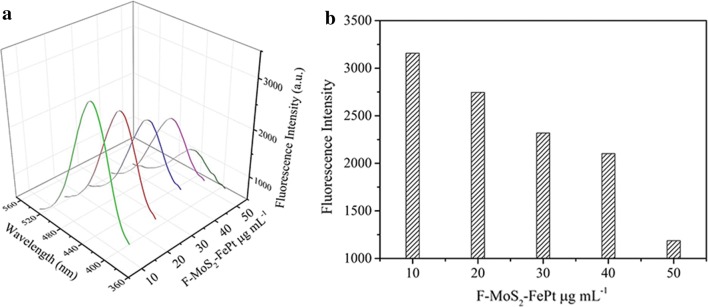
**a** Emission spectra of the *p*-Phthalic acid with series of different amount of F-MoS_2_-FePt NCs and constant concentration of H_2_O_2_. The reaction conditions are shown as follows: 5 mM TA, 0.25 M H_2_O_2_, along with different amount of F-MoS_2_-FePt NCs. **b** The corresponding fluorescence intensity of different amount of F-MoS_2_-FePt NCs

**Fig. 7:**
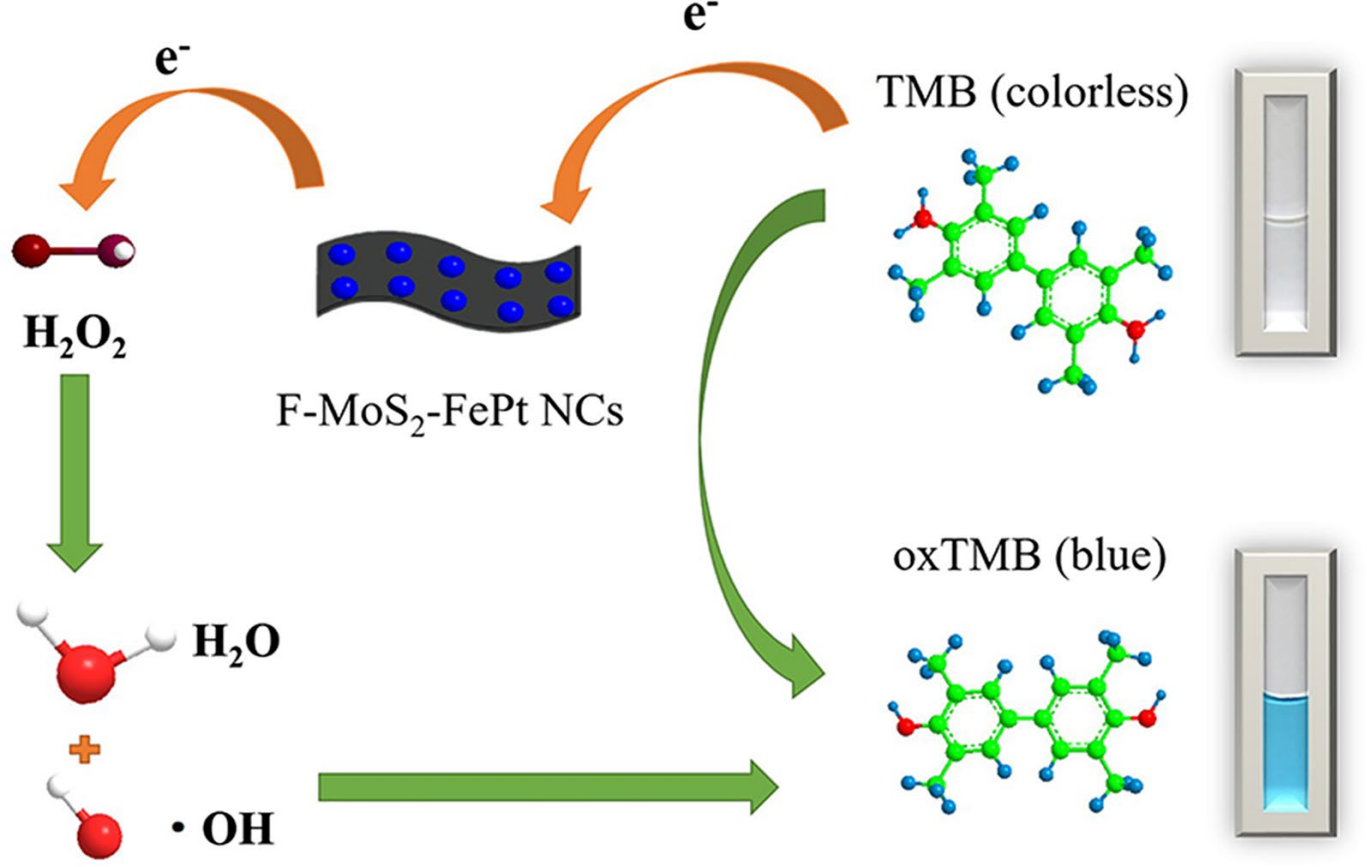
The catalytic mechanism of the constructed F-MoS_2_-FePt-TMB-H_2_O_2_ colorimetric sensing platform

Based on the prominent advantages such as cost-effective and high stability, p-Phthalic acid (TA) is applied to detect hydroxyl radicals (OH·) produced by the decomposition of H_2_O_2_. Fluorescence spectrometer is carried out to monitor the production generated from the combination of TA and hydroxide radical. As displayed in Fig. 6, as the amount of F-MoS_2_-FePt NCs varies from 10 to 50 μg mL^−1^, the fluorescence intensity decreases monotonically, which is caused by the reduction of hydroxide radical inhibited by high concentration of F-MoS_2_-FePt NCs. According to the literatures, electron transfer mechanism is applied to explain these catalytic activities, as shows in Fig. 7 [51–53]. F-MoS_2_-FePt NCs facilitate electrons shift between H_2_O_2_ and TMB. TMB can be easily absorbed on F-MoS_2_-FePt NCs, this is because the higher affinity (*K*_m_ = 0.4283) and donated lone-pair electrons from the amino groups cause the increase of the electron density and mobility on the NCs, as a result, the electron transfer to H_2_O_2_ is facilitated and the oxidation of TMB is speed up [54].

### Detection of H_2_O_2_ and sensing of the intracellular H_2_O_2_

**Fig. 8:**
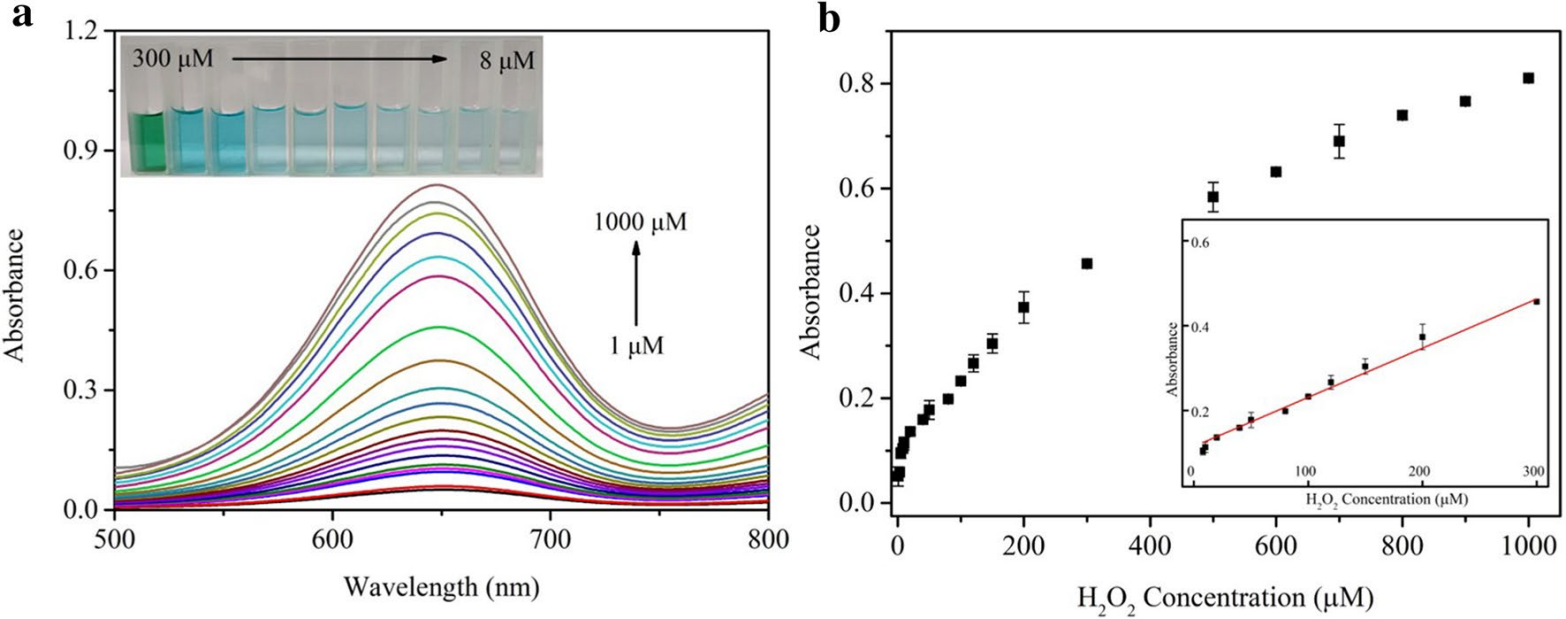
**a** The absorption spectra of H_2_O_2_ with various concentration and the corresponding images are shown in the inset picture. These experiments conditions are shown as follows: 1.4 mL CPBS (pH 4.2), 200 μL TMB (1 mM), 200 μL F-MoS_2_-FePt HNPs (20 μg mL^−1^) and different amount of H_2_O_2_. **b** Dose–response curve for H_2_O_2_ determination

The determination of H_2_O_2_ is carried out under the optimal conditions using UV–Vis absorption spectra ranging from 250 nm to 800 nm. As illustrated in Fig. 8a, with the increasing of H_2_O_2_ the UV absorption at 652 nm of the colorimetric system increases gradually. More importantly, the absorbance is in proportion to H_2_O_2_ concentration, providing a linear detection range from 8 to 300 μM (Fig. 8b). The corresponding image of the linear detection range of H_2_O_2_ is shown in the inset of Fig. 8a, which indicates that as the concentration decreases from 300 to 8 μM, the color of these solution turns from dark blue to baby blue, the detection limit is reckoned to be 2.24 μM. When compared with other nanomaterials-based colorimetric sensing platforms the linear detection range of the constructed sensing platform (F-MoS_2_-FePt-TMB-H_2_O_2_) is more wide and a lower limit of detection is obtained, as listed in Additional file 1: Table S1 [48, 50, 55, 56].

**Fig. 9:**
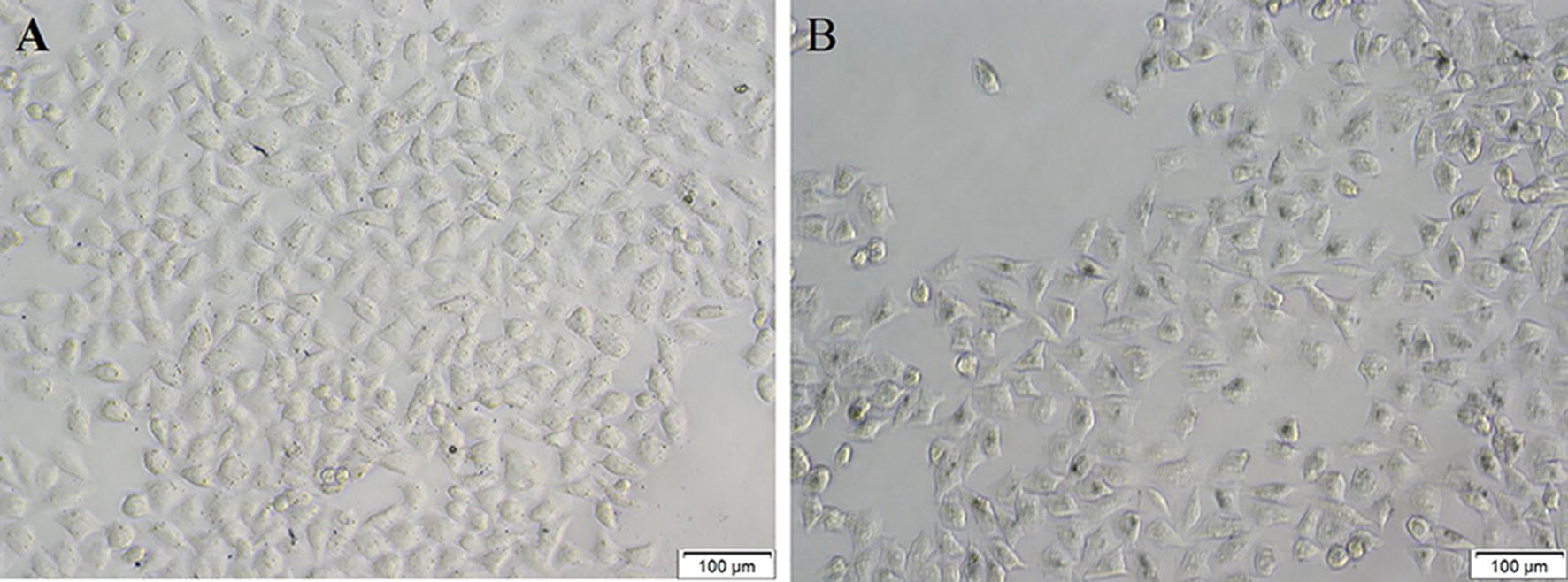
Images of MCF-7 incubated with F-MoS_2_-FePt-PEG-FA NCs and TMB (**A**) F-MoS_2_-FePt-PEG-FA and TMB and H_2_O_2_ (**B**)

To verify the feasibility of the detection of H_2_O_2_ in living cells, the obtained sensing platform is utilized to detect the intracellular H_2_O_2_. To improve the stability in the culture medium, the obtained NCs is modified with SH-PEG-FA. Then the established colorimetric sensing platform is further utilized to detect the intracellular H_2_O_2_ in MCF-7. After 4 h’ co-incubation with F-MoS_2_-FePt-PEG-FA, 0.2 mM TMB and 100 μM H_2_O_2_ are added into the plate and incubated for another 40 min. After co-incubation the cells are subjected to the electron microscope and the images are shown in Fig. 8. Compared with the cells only treated with NCs and TMB (Fig. 9A), the MCF-7 cells treated with NCs, TMB and H_2_O_2_ turn to clear blue (Fig. 9B). Without FA receptor on the cell membrane of normal cell (L02), nearly none NCs can be endocytosed (Additional file 1: Figure S5B). When treated with TMB only, MCF-7 remains colorless, as depicted in Additional file 1: Figure S5 A. The sensitive and selective of intracellular detection indicates that the F-MoS_2_-FePt-PEG-FA have the potential for monitoring of H_2_O_2_ in living cells.

## Conclusion

In this work, a sensitive and rapid colorimetric sensing platform for H_2_O_2_ detection utilizing F-MoS_2_-FePt NCs as artificial enzyme is constructed. The uniformly prepared FePt NPs are anchored on the surface of exfoliated few-layer MoS_2_ NSs by a facile operation. Series of experiments are carried out to verify the peroxidase-like catalytic activity of the obtained NCs. Under optimal conditions, the linear range of H_2_O_2_ detection is between 8 and 300 μM and the detection limit is 2.24 μM. Compared with other reported methods, F-MoS_2_-FePt NCs-based colorimetric sensing platform for H_2_O_2_ detection is a sensitive, simple and cost-effective method. To improve the stability and transmembrane performance of F-MoS_2_-FePt NCs, the surface of the prepared NCs is modified by SH-PEG-FA for intracellular H_2_O_2_ detection, which indicates that the sensor could be applied in living cells testing and has potential in disease diagnosis and therapy.

## Additional file

**Additional file 1: Figure S1.** The atomic force microscopy (AFM) images of the as-prepared Few-layers MoS_2_ nanosheets. **Figure S2.** The potential distribution of FePt-DMSA NPs. **Figure S3.** **a** TEM image of bulk MoS_2_ sheets; **b** HRTEM of MoS_2_-FePt. **Figure S4.** Image of FePt NPs before and after transferred from lipophilic to hydrophilic by DMSA via ligand exchange reaction. For the two phases, the upper layer is n-hexane, the lower is the water. **Figure S5.** **a** Images of MCF-7 cells incubated with TMB (1 mM) and **b** L02 cells incubated with F-MoS_2_-FePt-PEG-FA and TMB (1 mM). **Table S1.** Comparison of the linear range and the detection limit of H_2_O_2_ by means of different sensors.
