# Supplementary material for: A facile preparation of FePt-loaded few-layer MoS2 nanosheets nanocomposites (F-MoS2-FePt NCs) and their application for colorimetric detection of H2O2 in living cells
Source: J Nanobiotechnology. 2019 Mar 13;17:38. doi: 10.1186/s12951-019-0465-3 (PMC6417205; doi:10.1186/s12951-019-0465-3)
Supplement: Supplementary file 1 — Additional file 1: Figure S1. The atomic force microscopy (AFM) images of the as-prepared Few-layers MoS2 nanosheets. Figure S2. The potential distribution of FePt-DMSA NPs. Figure S3. a TEM image of bulk MoS2 sheets; b HRTEM of MoS2-FePt. Figure S4. Image of FePt NPs before and after transferred from lipophilic to hydrophilic by DMSA via ligand exchange reaction. For the two phases, the upper layer is n-hexane, the lower is the water. Figure S5. a Images of MCF-7 cells incubated with TMB (1 mM) and b L02 cells incubated with F-MoS2-FePt-PEG-FA and TMB (1 mM). Table S1. Comparison of the linear range and the detection limit of H2O2 by means of different sensors. [file 12951_2019_465_MOESM1_ESM.docx]

**Additional file**

**A facile preparation of FePt-loaded Few-layer MoS_2_ Nanosheets nanocomposites (F-MoS_2_-FePt NCs) and their application for colorimetric detection of H_2_O_2_ in living cells**

Zunfu Hu^a,b,c^, Zhichao Dai^b^, Xiaowei Hu^b^, Baochan Yang^b,d^, Qingyun Liu^d^, Chuanhui Gao^a^, Xiuwen Zheng^b *^, Yueqin Yu^a*^

a: Collage of chemistry and molecular engineering, Qingdao University of Science and Technology, Qingdao 266000, China

b: Key Laboratory of Functional Nanomaterials and Technology in Universities of Shandong, Linyi University, Linyi 276000, China

c: School of Materials Science and Engineering, Linyi University, Linyi 276000, China

d: School of Chemistry and Environmental Engineering, Shandong University of Science and Technology, Qingdao 266510, PR China

**Materials**： Pt(II) acetylacetonate (Pt(acac)_2_, 99%) were obtained from HWRK. Iron acetylacetonate (Fe(acac)_3_, 99%), 1,2-hexadecanediol, Di-n-octyl Ether, cis-1-Amino-9-octadecene (OAm, 90%), cis-9-Octadecenoic acid (OA, >85%), dimethyl sulfoxide (DMSO), DMSA, n-Butyllithium, TMB dihydrochloride hydrate (TMB), hydrogen peroxide (H_2_O_2_) and Molybdenum sulfide powder were offered by Aladdin Reagent Co. Ltd. All chemicals and solvents used were of analytical grade. MCF-7 (human breast cancer cell line) was provided by the Cell Bank of Chinese Academy of Sciences. L02 (human hepatocyte L02 cell line) was obtained from the American Type Culture Collection.

**Synthesis of FePt-DMSA.** 0.5mmol Pt (acac) _2_, 1mmol Fe (acac) _3_, and 3mmol 1,2-hexadecanediol accompany with 30 mL Di-n-octyl Ether were filled into a 100 mL three-necked bottle. The reaction mixture were magnetically stirred for 20 min under nitrogen atmosphere. The temperature was maintained at 100 ℃ for 20 min followed by 0.17 mL OAm and 0.16 mL OA. The temperature was pushed to 295 ℃ rapidly and kept for 1 hour. After cooled down to room temperature, 10 mL ethanol and 30 mL n-hexane were added into the bottle and centrifuged at 11000rpm. Repeat the procedure for three times. Then the obtained FePt NPs were dispersed in n-hexane for further application.

10 mg FePt NPs and 30 mg DMSA were mixed with 3 mL DMSO and ultrasound for 5 min. Then the mixture were shaking for 4 hours. After centrifuged for 5 min, the precipitate were washed with ethanol for three times.

**Preparation of Few-layer MoS_2_ nanosheets, MoS_2_-FePt HNPs and MoS_2_-FePt-PEG-FA.** 0.2 g molybdenum disulfide were reacted with 15 mL n-butyllithium under Ar atmosphere for 48 hours. After removing the unreacted n-butyllithium and residual solution, Ar saturated H_2_O was carefully dropped into bottle and the suspension was sonicated for 30mins, and then treated for 2 hours by the Ultrasonic cell crusher. The solution was centrifuged at 1500 rpm for 20 min, followed by 10000rpm for 30 min.

20mg MoS_2_ and 10mg FePt-DMSA were dispersed in 5ml water and sonicated for 20min to keep the temperature below 25℃ and centrifuged for 3 times to remove the unreacted MoS_2_ nanosheets. The obtained MoS_2_-FePt HNPs were resuspended in water.

30mg MoS_2_, 10mg FePt-DMSA and 10mg SH-PEG-FA were dispersed in 5 ml water and sonicated for 20 min to keep the temperature below 25℃ and centrifuged for 3 times to remove the unreacted MoS_2_ nanosheets and SH-PEG-FA. The obtained MoS_2_-FePt- PEG-FA HNPs were resuspended in water for further application.

**Characterization**

The morphology and structure of PB@FePt HNPs were obtained through the transmission electron microscopy (JEOL, JEM-2100, Japan) operated at 200 kV. X-ray diffraction (XRD) patterns were recorded in the 2θ range of 10–90 with a scan speed of 2° min-1 on a D8 Advance Powder using Cu Ka radiation (k = 0.1542 nm, 40 kV, 20 mA) and a proportional counter detector. In addition, Cary 4600 spectrometer (USA) was used to record the UV–vis spectra of different reaction systems. Fluorescence spectra were obtained by EDINBURCH FS5 spectrometer (US).

**Catalytic activity test.** Typically, the reactions were carried out in Citric acid buffer (CPBS, 0.2 M, pH=4.2) using different concentrations of MoS_2_-FePt NCs and 200 μL TMB (1 mM) with or without 200μL H_2_O_2_ (0.25 M). After 10 min, the enzyme steady-state kinetic experiments were performed on a UV–vis spectrophotometer in time course mode choosing H_2_O_2_ and TMB as substrates, respectively. The influences of pH values (2.2–8), temperatures (15–65°C) and the various concentration of NCs were investigated to evaluate the catalytic activity of MoS_2_-FePt NCs.

**Colorimetric Detection of H_2_O_2_**

In a typical procedure, TMB (200 μL, 1 mM) was oxidized in the presence of H_2_O_2_ with diffferent concentrations accompanied with MoS_2_-FePt NCs solutions (200 μL, 10 μg/mL) and sodium acetate buffer (1400 μL, pH 4.2). After reacting for 2 min, the resultant solution was used for adsorption spectroscopy measurement and the wavelength was set from 250 to 800 nm

**Intracellular H_2_O_2_ Detection**

2.0 mL MCF-7 cells (5 × 10^4^/mL) were seeded into a 6-well plate and cultured in DMEM medium with 10% fetal bovine serum (FBS) and 5% CO_2_ at 37 °C. After cultured for 24 h, the old medium was replaced by 2 mL fresh DMEM medium. Then, 100 μL MoS_2_-FePt-PEG-FA was added into the plate and maintained for 4 hours. The culture medium was discard and washed with PBS for three times. 2 mL fresh medium with 0.2 mM TMB and 100 μM H_2_O_2_ was added into the plate and cultured for 30 minutes. Finally, these cells were washed with PBS solution (pH 7.0) and took pictures with an inverted microscope.


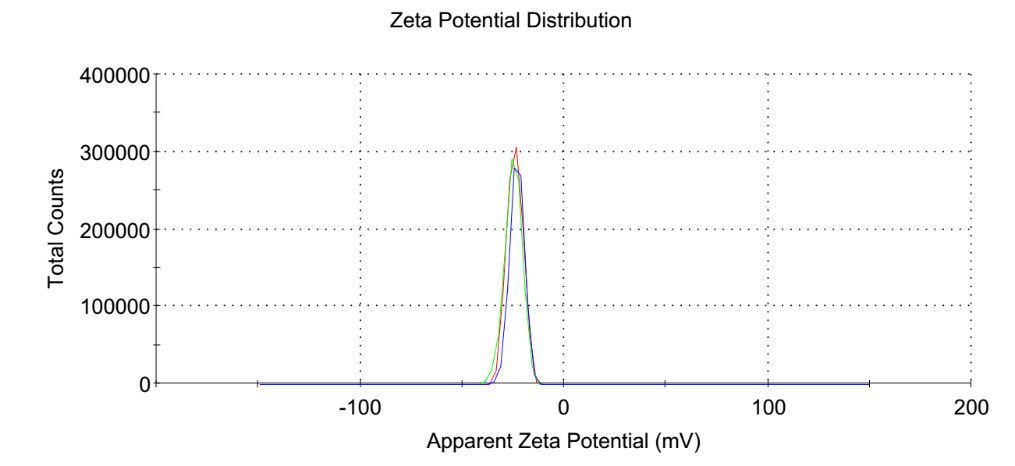

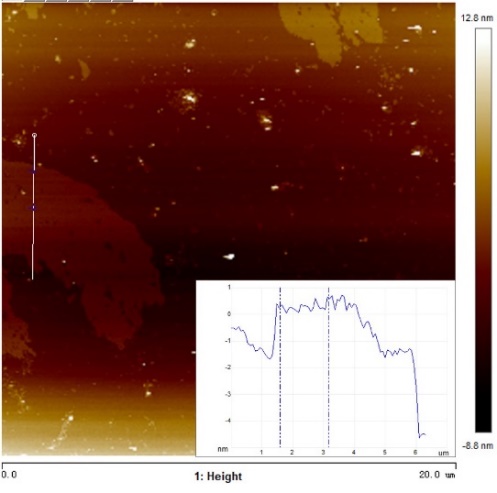
Figure S1. The Atomic force microscopy (AFM) images of the as-prepared ultrathin MoS_2_ nanosheets.


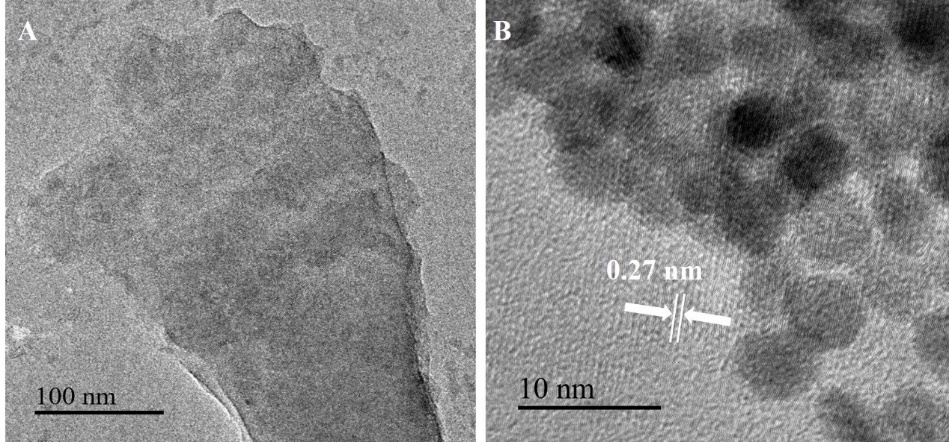
Figure S2. The potential distribution of FePt-DMSA NPs.

Figure S3. (A)TEM image of bulk MoS_2_ sheets; (B) HRTEM of MoS_2_-FePt.


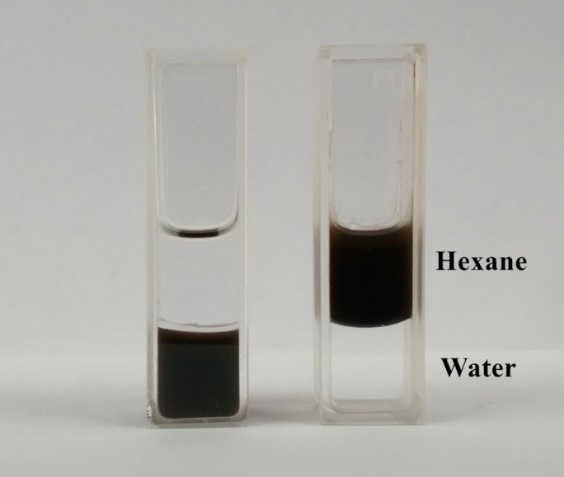
Figure S4. Image of FePt NPs before and after transferred from lipophilic to hydrophilic by DMSA via ligand exchange reaction. For the two phases, the upper layer is n-hexane, the lower is the water.

Table S1. Comparison of the linear range and the detection limit of H_2_O_2_ by means of different sensors.

| Catalysts | Linear Range | Detection Limit | Ref. |
| --- | --- | --- | --- |
| Cu_2_(OH)_3_Cl-CeO_2_ | 20-50 μM | 10 μM | [1] |
| Fe_3_O_4_@Cu@Cu_2_O | 4-50 mM | 2000 μM | [2] |
| GO-FeTPyP NCs | 20-500 μM | 72 μM | [3] |
| Au/Co_3_O_4_-CeOx | 10-1000 μM | 5.29 μM | [4] |
| CoS | 50-800 μM | 20 μM | [5] |
| Por-Ceria | 0.01-0.1 mM | 19 μM | [6] |
| N-G-Fe_3_O_4_ | 0-10 mM | 17.1 μM | [7] |
| NiPd HNPs  hNPs | 5-500 μM | 4.2 μM | [8] |
| F-MoS_2_-FePt NCs | 8-300 μM | 2.24 μM | This work |


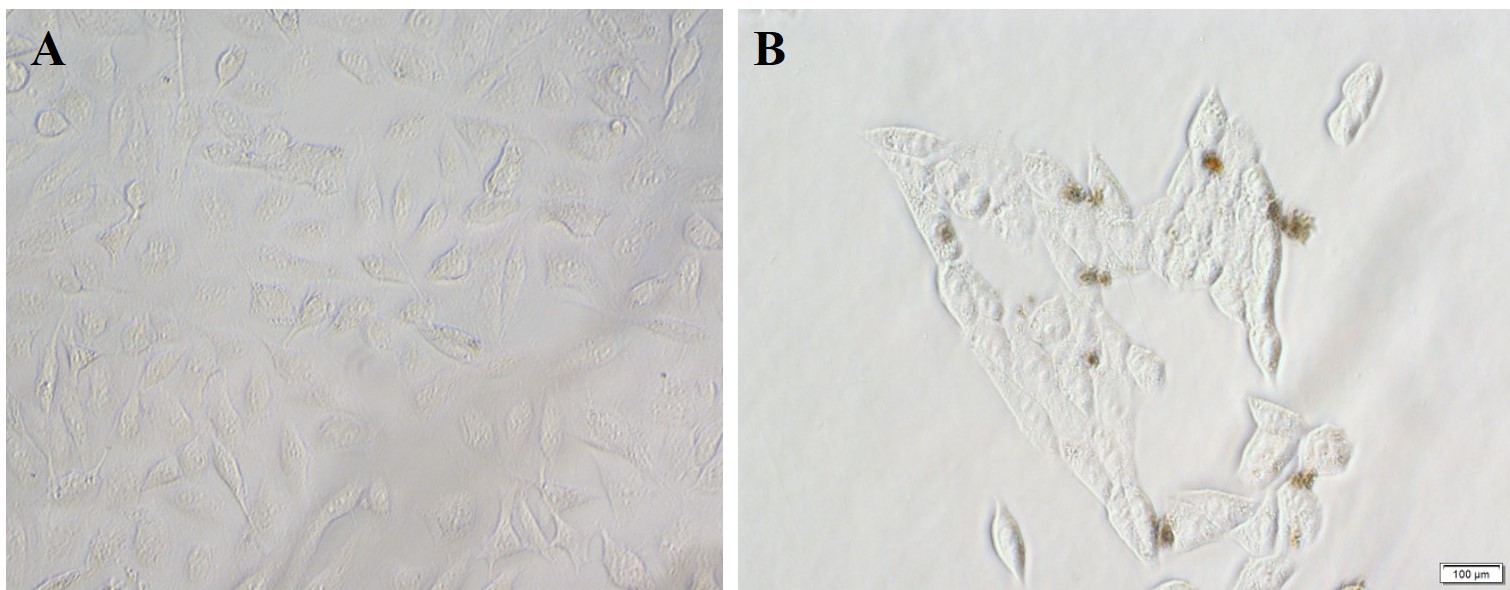
Figure S5. (A) Images of MCF-7 cells incubated with TMB (1mM) and (B) L02 cells incubated with MoS_2_-FePt-PEG-FA and TMB (1mM).

**References**

1.N. Wang,J. Sun,L. Chen,H. Fan,S. Ai,A Cu_2_(OH)_3_Cl-CeO_2_ nanocomposite with peroxidase-like activity, and its application to the determination of hydrogen peroxide, glucose and cholesterol. Microchim Acta 182 (**2015)** 1733–1738.

Z. Wang,M. Chen,J. Shu,Y. Li,One-step solvothermal synthesis of Fe_3_O_4_ @Cu@Cu_2_O nanocomposite as magnetically recyclable mimetic peroxidase. Journal of Alloys and Compounds 682 (**2016)** 432-440.

C. Socaci,F. Pogacean,A. R. Biris,M. Coros,M. C. Rosu,L. Magerusan,G. Katona,S. Pruneanu,Graphene oxide vs. reduced graphene oxide as carbon support in porphyrin peroxidase biomimetic nanomaterials. Talanta 148 (**2016)** 511-517.

H. Liu,Y. Ding,B. Yang,Z. Liu,Q. Liu,X. Zhang,Colorimetric and ultrasensitive detection of H_2_O_2_ based on Au/Co_3_O_4_ -CeO x nanocomposites with enhanced peroxidase-like performance. Sensors and Actuators B: Chemical 271 (**2018)** 336-345.

[71]. H. Yang,J. Zha,P. Zhang,Y. Xiong,L. Su,F. Ye,Sphere-like CoS with nanostructures as peroxidase mimics for colorimetric determination of H_2_O_2_ and mercury ions. RSC Advances 6 (**2016)** 66963-66970.

Q. Liu,Y. Yang,X. Lv,Y. Ding,Y. Zhang,J. Jing,C. Xu,One-step synthesis of uniform nanoparticles of porphyrin functionalized ceria with promising peroxidase mimetics for H_2_O_2_ and glucose colorimetric detection. Sensors and Actuators B: Chemical 240 (**2017)** 726-734.

[20]. W. Zhang,C. Chen,D. Yang,G. Dong,S. Jia,B. Zhao,L. Yan,Q. Yao,A. Sunna,Y. Liu,Optical Biosensors Based on Nitrogen-Doped Graphene Functionalized with Magnetic Nanoparticles. Advanced Materials Interfaces 3 (**2016)** 1600590-1600595.

[31]. Q. Wang,L. Zhang,C. Shang,Z. Zhang,S. Dong,Triple-enzyme mimetic activity of nickel-palladium hollow nanoparticles and their application in colorimetric biosensing of glucose. Chem Commun (Camb) 52 (**2016)** 5410-5413.
